# Lab-on-a-chip: an advanced technology for the modernization of traditional Chinese medicine

**DOI:** 10.1186/s13020-024-00956-4

**Published:** 2024-06-09

**Authors:** Zenghui Lu, Yue Yuan, Qiang Han, Yu Wang, Qionglin Liang

**Affiliations:** 1 Institute of Traditional Chinese Medicine-X, State Administration of Traditional Chinese Medicine Third-Level Laboratory of Traditional Chinese Medicine Chemistry, Modern Research Center for Traditional Chinese Medicine, Tsinghua University, Beijing, 100084 China; 2 Beijing Key Laboratory of TCM Pharmacology, Xiyuan Hospital, China Academy of Chinese Medical Sciences, Beijing, 100730 China

**Keywords:** Microfluidic chip, Pharm-lab-on-a-chip, Traditional Chinese medicine, Quality control, Active ingredient screening, Compatibility of traditional Chinese medicine

## Abstract

Benefiting from the complex system composed of various constituents, medicament portions, species, and places of origin, traditional Chinese medicine (TCM) possesses numerous customizable and adaptable efficacies in clinical practice guided by its theories. However, these unique features are also present challenges in areas such as quality control, screening active ingredients, studying cell and organ pharmacology, and characterizing the compatibility between different Chinese medicines. Drawing inspiration from the holistic concept, an integrated strategy and pattern more aligned with TCM research emerges, necessitating the integration of novel technology into TCM modernization. The microfluidic chip serves as a powerful platform for integrating technologies in chemistry, biology, and biophysics. Microfluidics has given rise to innovative patterns like lab-on-a-chip and organoids-on-a-chip, effectively challenging the conventional research paradigms of TCM. This review provides a systematic summary of the nature and advanced utilization of microfluidic chips in TCM, focusing on quality control, active ingredient screening/separation, pharmaceutical analysis, and pharmacological/toxicological assays. Drawing on these remarkable references, the challenges, opportunities, and future trends of microfluidic chips in TCM are also comprehensively discussed, providing valuable insights into the development of TCM.

## Introduction

With the remarkable success of traditional Chinese medicine (TCM) in combating the COVID-19 pandemic, an increasing number of individuals are turning to this ancient yet potent form of medicine for treatment, not only in Asia but across the globe [1]. Adhering to the theories and principles of TCM, clinical experts employ precise TCM formulas tailored to individual patients, guided by diagnostic outcomes and the combination principles of Chinese medicine.

The unique efficacy of TCM stems from its distinctive properties. Firstly, the pharmacological and toxicological effects of TCM have been extensively scrutinized over centuries of clinical cases, demonstrating unparalleled biocompatibility owing to its natural composition derived from plants, animals, and minerals. Secondly, the extensive literature on TCM has documented its multi-target effects, which clinical experts adeptly harness for treatment. Thirdly, a noteworthy characteristic of TCM's practical application is the classification of various species or multiple sources of the same species under a single category, particularly in the realm of herbal-based TCM. These properties exemplify the phenotypic traits of compounds present in TCM, with chemical substances serving as the vital focal point of TCM research.

To heritage and fully unlock the potential of TCM, numerous systematic research endeavors have been undertaken. These efforts have ranged from identifying the constituents of TCM to studying its effects at the molecular, cellular, and organismal levels. Scientists have made significant progress in various disciplines related to TCM, including quality control, compound separation, and pharmacodynamic substance screening. However, unlike conventional pharmaceuticals, understanding the underlying theories of TCM presents a fundamental challenge. To address this, researchers have introduced and proposed a range of classical and cutting-edge technologies and approaches within the field. For instance, fingerprint analysis [2] and Q-markers [3] have been employed for quality control purposes. Meanwhile, systems biology [4], network pharmacology [5], and chinmedomics [6] have been utilized to unravel the intricate interactions between TCM and the human body. These groundbreaking advancements have consistently stimulated scientists to explore innovative ideologies, strategies, and methodologies, thereby driving the progress of TCM through the integration of novel technologies.

Microfluidic technology is centered around the manipulation and utilization of microflow through microtubules and valves embedded in microdevices. This manipulation of droplets and laminar flow on chips simplifies research processes focused on intermolecular recognition and separation. The advantages of working at the microscale enable precise control and management of reactions at the molecular level, such as separating trace substances from complex mixtures. Additionally, microfluidics provides flexibility in terms of liquid composition, flow rate, temperature, and routing, thus contributing to diverse mechanical properties. These capabilities allow for the creation of in vitro microenvironments that mimic cellular and organ-level conditions in living organisms. Cells-on-a-chip and organoids/organs-on-a-chip have emerged as breakthrough technologies, surpassing the limitations of conventional culture methods and accelerating our understanding of the fundamental principles of life sciences [7]. Microfluidics also offers modularity and expandability, making it a robust platform for integrating chemistry, biology, and biophysics technologies. The design of each part or chamber on a microfluidic chip determines its functionality, and the integration of different components is facilitated by microtubes. This integration has allowed for the successful employment of advanced analytical chemistry techniques in conjunction with cells/organs/organoids-on-a-chip platforms. For example, mass spectrometry has been coupled with these platforms to observe metabolites [8], while the coupling of affinity vectors enhanced the isolation efficiency of targeted proteins/cells [9]. Given the exceptional performance of microfluidic chips, a novel paradigm known as Pharm-on-a-chip has been proposed for drug analysis and evaluation [10].

**Fig. 1:**
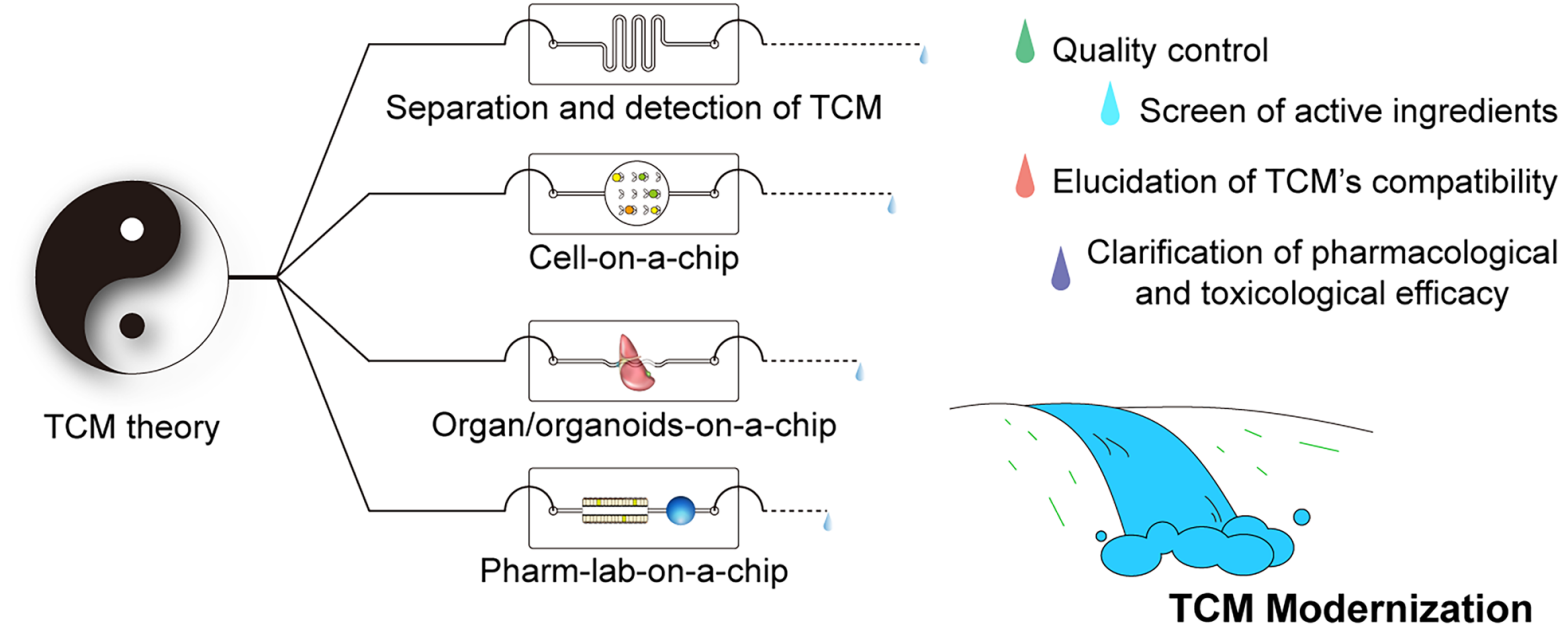
Illustration of this review profile

In this paper, our focus lies on the advanced development of microfluidics, particularly in the context of TCM. We provide a comprehensive summary and illustration of the novel aspects and advantages of microfluidic technologies, ranging from their inherent features to the representative demonstrations of microfluidic chips. We discuss the existing obstacles and the potential applications of microfluidics in TCM. Furthermore, we present opportunities and future trends that highlight the potential of microfluidics in contributing to the modernization of TCM, thereby providing updated insights into this field (Fig. 1).

## The modernization of traditional Chinese medicine

### Classical theories and typical characteristics of TCM

While the foundation of constructing TCM theories lay in the healing effects of diseases, the mechanism description of TCM was derived from eastern classical philosophy, which set it apart from chemical medicines that relied on anatomical physiology [11]. TCM consists of plants, animal products, and inorganic salts. Unlike chemical drugs, which need to undergo separation and purification processes before they can be administered, the majority of TCM (e.g., *wan* (pill) [12], *san* (powder) [13], or *tang* (decoction) [14]) is typically prepared with minimal processing. Oral administration was the classical route of administration, and thus, the taste properties (sour, bitter, sweet, acrid, salty, etc.) were considered one of the properties of TCM [15]. With the accumulation of clinical cases, the systematic theories of TCM have been cultivated and developed. The effects of TCM are categorized into *four natures*: hot, warm, cool, and cold. For example, TCM includes multiple types of antidiarrheal drugs, each exerting different effects on symptoms and the intestinal bacterial community despite all being used to treat diarrhea [16]. The beneficial efficacies of TCM are summarized in terms of various treatment outcomes, such as *qi-tonifying* and *drying dampness*, which have facilitated clinical applications [17, 18]. These concise descriptions were closely related to the organic functions of the human body. When observed at the molecular, cellular, and organ levels, numerous intricate mechanisms can be uncovered that underlie these individual efficacies.

Interestingly, complex interactions existed among different TCM components, commonly called the concept of TCM compatibility. Appropriate combinations would trigger synergistic effects, whereas inappropriate combinations might lead to side effects or toxicity [19, 20]. In TCM prescriptions, each component plays a specific role, and the theory of *monarch/minister/assistant/guide* has been applied to depict the primary and secondary relationships among these components. The compatibility of TCM has long been distinguished from the linear signal cascade illustrated by pharmacology due to the lack of molecular targets and pathway networks during its birth and formation. As a result, there is an urgent need for clarification in this regard.

### Frontiers of TCM modernization

The modernization of TCM aims to preserve, clarify, and promote the theories and practices of this ancient healing system. This involves enhancing research, exploration, production, management, and application of modern TCM by integrating traditional features and leveraging modern scientific technologies [21, 22]. Over the past three decades, this strategy has been in place, encompassing diverse domains relevant to TCM, such as genetic resources, quality control, new drug development, and industrialization. Furthermore, since the 18th National Congress of the Communist Party of the People's Republic of China, approximately 30 policies and measures have been implemented to facilitate the modernization of TCM [23].

Given multiple production areas or species within TCM, proper production methods are crucial for maintaining drug efficacy. Molecular discrimination of natural medicine facilitates a deeper understanding of TCM [24]. In original identification, DNA barcodes, including *matK*, *rbcL*, ITS, and ITS2, were exploited in medicinal plant materials [25]. The strategy based on DNA metabarcoding combined with minibarcode enables precise identification of the origin of TCM. Importantly, this novel strategy can reveal distinct genetic variations in TCM that contribute to changes in chemical components and biological activity, though the morphological characteristics of the wild source medicine show no apparent differences [26, 27].

In chemical characterization, the evaluation and confirmation of marker compounds that reflect typical pharmacology are essential but pose obstacles, especially when dealing with multiple active structural analogs [28]. A rational method called quantitative analysis of multi-components by a single marker (QAMS) was proposed and accepted by researchers [29]. The QAMS strategy simplifies the quality control process by establishing the international relationship between major active components and other effective ingredients [30]. However, due to limitations in quantitating multiple pieces or dealing with structural analogs, the holistic evaluation strategy known as fingerprinting has been further applied in TCM to capture comprehensive quality information [2]. The standard decoction provides detailed information about the processing, extraction, and production elements, ensuring a standardized analysis procedure and improving repeatability [14]. Given the multiple processes involved in wild natural medicine, several factors can affect its efficacy and safety. A novel concept and procedure called Q-marker, which focuses on the entire procedure and biological efficiency, has been applied in the evaluation and monitoring of TCM [3].

The clarification and characterization of the efficiency mechanism of TCM remained difficult, necessitating innovative theories to drive the modernization of TCM. In response to the urgent need for elucidating the combination theory of TCM, Traditional Chinese Medicine Chemomics (TCMC) was proposed and established its historical significance in the modernization of TCM [4, 31]. TCMC aims to gradually discover and confirm the composition of prominent chemical substance groups within the overall chemical substance group of the compound. This is achieved by analyzing the interplay between chemical information flow and multi-parameter biological information flow across different levels of chemical substance groups. According to the perspective of TCMC, the compound of TCM, as an overall chemical substance group, can be seen as an organic combination of several related sub-chemical substance groups, including the global chemome, effective chemome, and effective constituent group. These sub-chemical substance groups manifest in two primary forms: medicinal materials and components (also known as effective components) that represent specific characteristics of the compound. Hence, in this sense, the compatibility of effective compositions and the combination of TCM are essentially aligned [32].

Key features reflecting synergy or antagonism were generated from the global chemome of TCM, the effective chemome, and the effective constituents group. To distinguish these ingredients, spectrum-effect relationships were used to link biological effects and pharmacodynamic data [33]. In the case of the antioxidant composition of *Lycii Fructus*, also named *Gou qi*, the regression coefficient (> 0) between compounds and activities was operated. The effective constituents were identified and confirmed using standards and UPLC-MS/MS, partially confirming the effective chemome [34]. System pharmacology focuses on holistic profiling and biological processes, providing a more comprehensive reflection of changes in physiological or pathological conditions [4, 35]. For instance, metabolomics has thrived in TCM research, covering various metabolites ranging from lipids and bile acids to amino acids [36–38]. Based on system biology and serum pharmacochemistry of TCM, Chinmedomics was proposed and applied in the identification of biomarkers of syndromes, evaluation of effectiveness, and discovery of pharmacodynamic material basis of herbal medicines/herbal formulas [39]. Dampness-heat jaundice syndrome-induced metabolic disorders, including glycerophospholipid metabolism, arachidonic acid metabolism, and glycosylpho-sphatidylinositol-anchor biosynthesis. Yin-Chen-Hao-Tang, one of the conventional TCM formulas, has shown effectiveness by adjusting these abnormal metabolic processes [40]. Network pharmacology is a powerful tool for predicting molecular targets and analyzing signal pathways of TCM. Its widespread application has improved high-throughput virtual screening of TCM [41–43]. In vivo*,* processes of TCM are monitored over an extended period to confirm effectiveness and safety. Combining in silico predictions with objective research validations is conducive to identifying effective constituent groups. Integrative pharmacology-based traditional Chinese medicine (TCMIP) focuses on constructing and evaluating multi-dimensional associations among the chemical and ADME/PK profiles of TCM, the disease-syndrome-formula association network, and pharmacological actions. This approach allows for qualitative and quantitative assessment of the PK-PD correlation in vivo [44].

### Challenges and opportunities of modernizing TCM

**Fig. 2:**
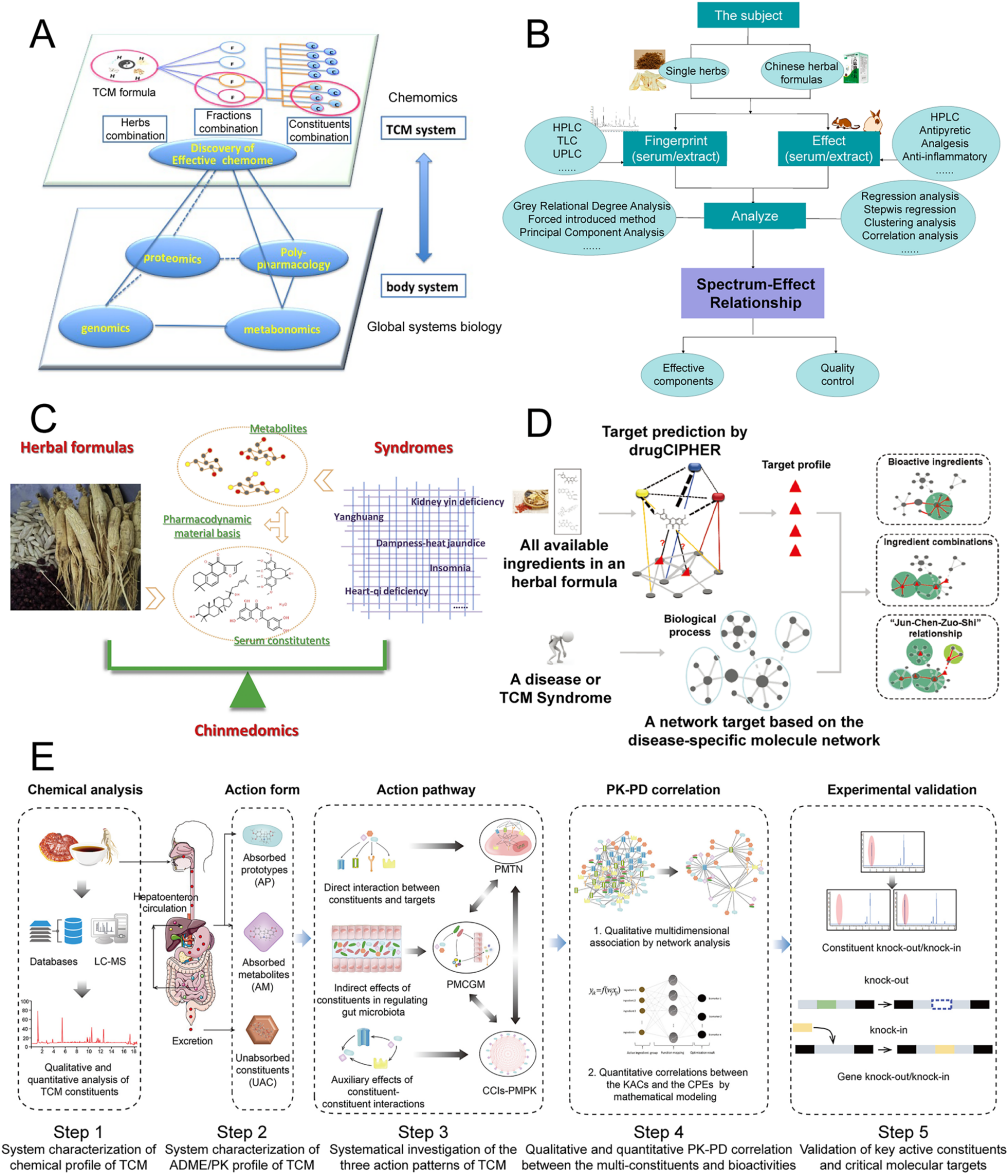
Some advanced theories and strategies in characterizing TCM’s efficacy mechanism. **A** Traditional Chinese medicine chemomics [4]; **B** Spectrum-effect relationships [33]; **C** Chinmedomics [39], Copyright, 2020, Elsevier; **D** Network pharmacology [43], Copyright, 2013, Elsevier; **E** Integrative pharmacology-based TCM [44]

Since the release of the modernization of TCM, standardized and objective evaluation methods have been developed and applied to quality control, pharmacological/toxicological assessment, and industrialization. One crucial aspect of TCM modernization has been clarifying classic theories, including but not limited to *four natures*, *five states*, *channel tropism,* and compatibility of TCM. Throughout the modernization process of TCM, various theories and modes of illustration have been proposed and acknowledged. These include the exploration of active compounds, pharmacodynamics, biological responses, and synergistic/antagonistic effects (Fig. 2). The analysis of TCM theories has revealed different facets of this ancient practice, highlighting its unique qualities as a multi-component and multi-target approach. These characteristics distinguish TCM from conventional chemical drugs, which typically either originate from natural sources or are artificially created in a laboratory setting. The integration of novel technologies has accelerated modernization of TCM. For instance, chemomics and systematic pharmacology have played a significant role in the development of TCMC [4], while the advancements in metabolomics have laid a solid foundation for the field of Chinmedomics [39].

Considering that TCM and biological individuals are separate systems, the clarification of effective chemome and the constituents group of TCM relies on the characterization pattern of effective constituents and their physical effects. However, the lack of a multi-dimensional monitoring and analysis protocol that can simultaneously assess multiple cells, organoids, or organs hinders the understanding of compatibility mechanisms between two or more different TCMs. Microfluidics has emerged as a thriving functional platform for life research due to its excellent microfluidic features and powerful integration [45]. Microfluidic chips are designed to manipulate microfluids or droplets through various channels and chambers. These chips offer advantages in terms of cell, organoid, and tissue growth, minimizing the limitations of conventional life culture and biological assays [46]. Notably, the laminar flow feature and the successful co-culture of multiple cells and organoids on chips hold promise in elucidating complex interactions between different organs. This approach is beneficial for characterizing TCM compatibility.

## Microfluidics and microfluidic chips

### Features and phenomenon of microfluidics

Microfluidics is a sub-discipline of fluid mechanics that focuses on the science of fluids at the micrometer scale. Unlike macroscopic fluidic systems with turbulent flow, microfluidics operates primarily under laminar flow conditions, with surface tension becoming non-negligible [47].

**Fig.3:**
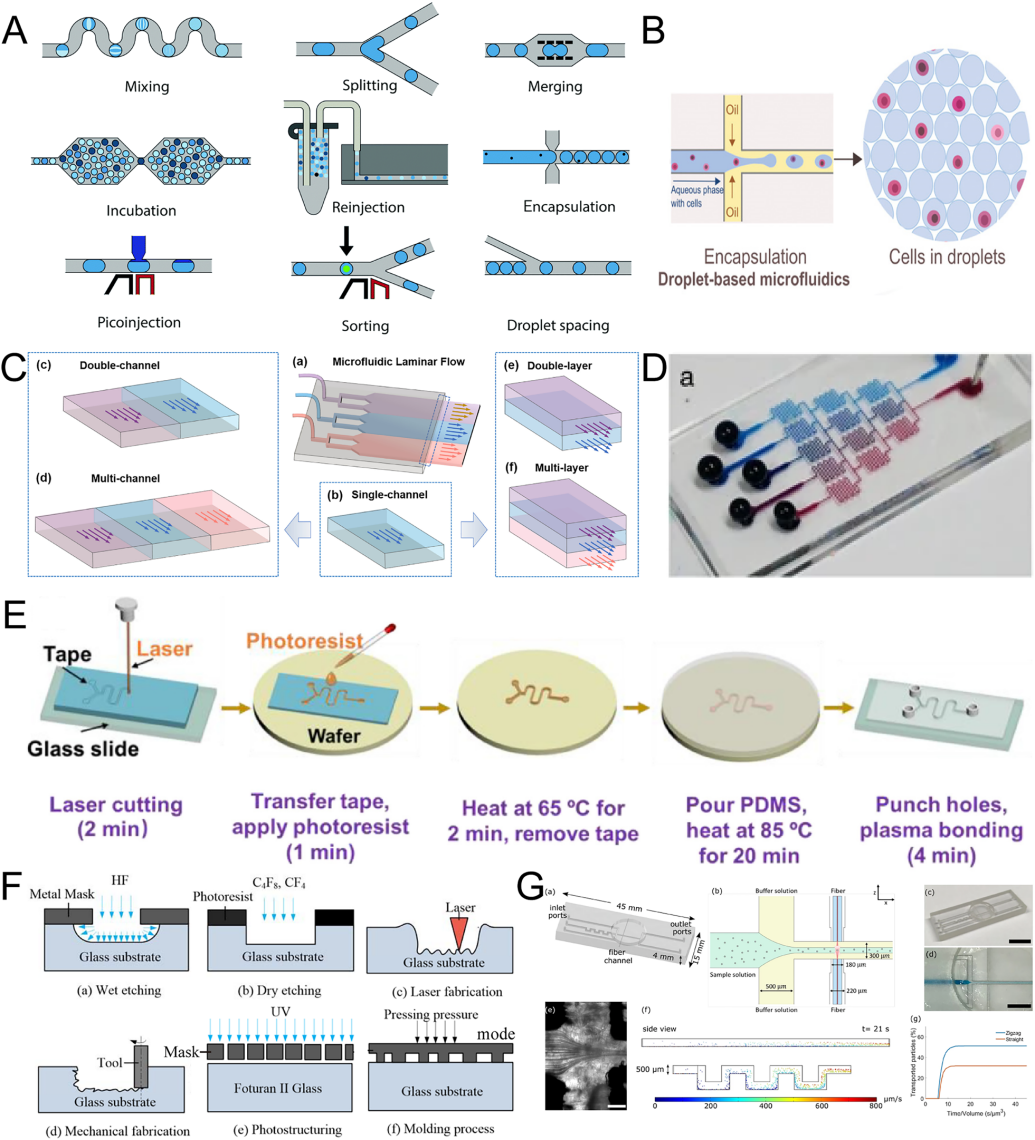
Typical structures and fabrication of microfluidics. **A** Droplet simulation [51]; **B** Droplet microfluidics for single cell analysis [65]; **C** Laminar flow on chips [66], Copyright, 2022, Elsevier; **D** Christmas tree model [67]; **E** PDMS chip fabrication [68], Copyright, 2022, Elsevier; **F** Six typical glass microstructure fabrication techniques [55]; **G** 3D Printed microfluidic chip [69]

In a tube where two or more flows are mixed, the state of the mixed flow depends on the Reynolds number (Re), which is directly proportional to the fluid velocity and inversely proportional to the intensity of the viscous friction. When Re is less than 2000, the flow is laminar, and mass transfer between layers is mainly due to diffusion. Conversely, when Re is greater than 3000, the flow is turbulent. In microfluidics, the typical state of the flow is laminar, with Re ranging from 10^–2^ to 10^–3^ [48]. By utilizing tailored microchannels, laminar flow can be maintained at scales typically absent in macro-scale flows. This feature facilitates micro-length scale flow research and potential applications (Fig. 3C, D).

Surface effects are crucial in microfluidics due to the high surface-to-volume ratios involved. Surface tension arises from the imbalanced interactions between molecules at the material's surface. Generally, strong polar liquids exhibit higher surface tension than nonpolar liquids, and surface tension can be reduced through chemical modifications such as fluorination. As the temperature of the fluid increases, surface tension decreases due to enhanced Brownian motion of solvent molecules. The mechanical imbalance on the liquid surface drives the formation of drops, with smaller diameter drops exhibiting higher surface tension. From this principle, droplet microfluidics has been developed as an analytical technique that manipulates mutually immiscible phases within a microchannel (Fig. 3A, B) [49–51].

Additionally, operating at the micro-scale leads to a significant reduction in the mass/volume of chemical and biological reagents, a phenomenon known as the scaling law. When a system decreases in size proportionally in all dimensions, the volume of the system will decrease cubically.

### Construction of microfluidic chips

Closed or semi-closed structures, such as micro-tubes, microneedles, and microfluidic chips, serve as standard platforms for microfluidics researchers [52, 53]. Rigid materials ranging from glass to silica were previously exploited as the materials of chips for their optical properties, good insulating properties, high resistance to mechanical stress, high surface stability, and high solvent compatibility. The fabrication involved photolithography and wet etching protocols (Fig. 3F) [54, 55]. Computer software such as AutoCAD was employed to design the devices on different slides, and chemical etching [56, 57], laser etching [58], and fused operation [59] are viable methods for constructing designed patterns consisting of different channels, holes and chambers on glass/silica chips [60–62]. Surface modification offers the advantage of creating smoother bottom surfaces, reducing residues from bio-samples, and improving the optical properties of the chips [55, 63]. Hydroxylation is commonly achieved using a piranha solution, and plasma treatment and silanization reagents are used for surface hydrophobic modification. Compared with glass chips, silica chips offer advantages in applications related to refraction [64].

The solid materials were complex to fabricate sophisticated channels and chambers. Polymerics including polydimethylsiloxane (PDMS), polymethyl methacrylate, and cyclic olefin copolymer were employed to construct microfluidic chips due to their optical transparency, mouldability, and cost-effectiveness. Among them, PDMS is the most commonly used material. Cured PDMS exhibits biocompatibility and allows for the permeation of liquids and gases, making it suitable for in vitro tissue culture. Constructing PDMS chips through soft lithography involves two essential procedures [70, 71]. Firstly, a stamp (referred to as a master mold) with specific channels, chambers, or other complex structures is fabricated through a photography process. Photosensitive materials deposited on substrates can be classified as positive or negative photoresists. Following exposure, the pattern remains in the case of negative photoresist, while it dissolves in the case of positive photoresist. Secondly, a mixture of PDMS and curing agent is poured into the obtained stamp, and polymerization is induced by heat (Fig. 3E). Benefited from the functional group of PDMS, the peeling-off polymer was usually bonded in glass/silicon, and there were many hybrid chips, including glass-PDMS [72], silico-glass [73], sandwich (glass-PDMS-glass) [74]. The limitation of PDMS (e.g., weak chemical compatibility, absorption of molecules) presented opportunities for surface modification of PDMS or other materials on chips [75]. For example, with the coating layer formed by tetraethoxysilane and methyltriethoxysilane, the chemical resistance of the PDMS chip was improved significantly [76, 77]. Additionally, 3D printing has been increasingly exploited for fabricating microfluidic chips and was regarded as one of the most promising construction methods (Fig. 3G) [69].

The manipulation of multi-stream flows within microchannels is facilitated by strategically designed junctions, allowing for precise control following the introduction of liquid into the chip via inlet ports [66, 78]. The multi-stream laminar flow on a chip has the capacity to generate varying concentration of mixture, including linear and nonlinear gradients (e.g., bell-shaped) [79]. It finds widespread application in the preparation of drugs with diverse concentrations or compositions. T-shaped junction was employed to encapsulate molecules or cells within single droplets, thereby serving as a powerful tool for single-cell analysis (Fig. 3A–D). Moreover, the coaxial micro flow was accessible and the insoluble liquids fluid could be tailored to meet specific functional requirement, such as replicating the physiological characteristics of blood vessels in vitro [80]. In certain circumstances, the presence of an effective fluid mixing system becomes imperative. Microfluidic mixer chips were engineered with interconnected multi-channel networks, incorporating hybrid units comprising annular channels. These channels divide the input flow into smaller segments, which were subsequently chaotically reorganized [81]. Beyond biomedical applications, multi-stream flow could also be used in advanced materials and sensing, such as the formation of particles with varying complexity and heterogeneity [82, 83]. These instances underscored the sophisticated nature of microfluidic chips, emphasizing the necessity for researchers to design chip structures according to their research objectives and leverage unique fluidic effects to their fullest potential.

Microfluidics were also applied in cell patterning that was a significant technique for biological studies and tissue engineering [84, 85]. Lift-off cell lithography, for instance, utilized a poly-l-lysine coated glass coverslip, onto which a 4% polyvinyl alcohol sacrificial layer was spin-coated. Through the use of SU-8 3005 and photolithography, a 5 μm thin film with porous matrix was fabricated above poly alcohol layer, which was subsequently etched using O_2_ plasma etching. Reapplication of a poly-l-lysine coating onto the chip’s surface completed the construction of micro devices. This novel chip design, with its poly-l-lysine coating, was conducive to cells adhesion through electrostatic attraction. Following the dissolution of the polyvinyl alcohol layer, cells adhering to the SU-8 3005 file were released from the chip. Consequently, an array of cells was formed and various patterning cells were produced by different micro-patterned [86]. Furthermore, three-dimensional cell manipulation was realized through the establishment of electric fields by multiple scaffold structure. PDMS and nano-sized carbon powder were chosen to create an electrically conductive sheet, onto which a 100 μm thick insulating layer of PDMS was cast on one side of the sheet. The machined voltage and ground scaffold layers were then stacked and bonded together to construct the multiple scaffold structure. When a voltage input was applied, different types of cells became polarized and were attracted towards the interior part of the scaffold structure. This novel method enabled the operation of 3D cellular patterns within the 3D domain by dielectrophoresis [87].

### Separation and detection of drug

The separation of drug molecules related to multiple substance exchange procedures, including liquid–liquid, solid–liquid and gas–solid migration. For example, triterpenoid saponins were extracted and confirmed from *n*-butanol extraction of *Aesculus chinensis* Bge [88]. Thin layer chromatography, combined with reference herbs, has historically been utilized for rapid verification and preliminary quality assessment of TCM. Moreover, employing imaging technology has enabled the discrimination of species from multiple botanical origins, such as *Fritillaria* Bulbus and *Fritillariae Cirrhosae* Bulbus, using a high performance thin layer chromatography system [89]. Column chromatography remains a classical method for separating and purifying novel compounds in TCM, with macroporous resin and silica gel frequently employed as stationary phases [90]. The advent of high performance liquid chromatography (HPLC) has significantly enhanced the separation capacity of TCM. In a typical reverse-phase separation system utilizing a C18 column, chemical ingredients from crude TCM extracts could be efficiently separated and detected within an hour and a half [91]. This analytical system was further optimized through the use of special stationary phases and compatible solvents, rendering it applicable to analysis of numerous components of TCM. Additionally, gas chromatography (GC) has been explored for the detection of low polarity constituents, particularly volatile substances, in TCM [92].

The analysis process of TCM is hindered by various factors, including the wide array of compounds, inconsistent content, and complex matrix. This challenge promotes technological integration, with HPLC/GC usually participated in the development of novel analytical methods due to their exceptional separation capabilities. For constituents containing chromophores (e.g., flavonoids, anthraquinones), ultraviolet and visible spectrum analysis, known as HPLC–UV, was commonly employed in TCM quality control, with various analytical methods documented in the Chinese Pharmacopoeia. Conversely, constituents lacking chromophores (e.g., diterpenoid alkaloid, triterpenoid saponins) necessitate post-column analysis using evaporative light scattering detectors and charged aerosol detectors in conjunction with HPLC [93, 94]. The widespread adoption of mass spectrometry has significantly advanced the analysis process and strategy of TCM [95]. HPLC–MS, characterized by its high throughput and sensitivity, matched the demands of in vivo/in vitro TCM analysis. In nondestructive TCM analysis, nuclear magnetic resonance spectroscopy and infrared spectrum were accessible. For instance, infrared spectrum were applied in the real-time monitor of Chinese medicine injections or oral liquid formulations [24, 96].

Chromatography, for instance, enables rapid and effective separation, while HPLC–UV provides accurate and non-destructive compound analysis. Additionally, HPLC–MS offers high throughput and sensitivity for qualitative and quantitative evaluations. Despite these achievements, these analytical processes often suffer from resource-intensive procedures, limited biocompatibility, and labor-intensive requirements.

Operating analytical procedures for drugs in microfluidics has the advantage of reducing the consumption of biological samples and reliance on costly and complex instruments. The continuous flow also puts momentum into liquid–liquid extraction [97]. Immiscible solvents were used to dissolve and extract drug compounds, respectively. On a three-phase microfluidic chip, the crude extraction was dissolved in the basic aqueous phase, and the organic phase was used to extract medical constituents [98]. After the purification procedure of acid aqueous treatment on the chip, strychnine and brucine were obtained from plant extraction [99]. Successive laminar flow extraction has been found to enhance the diffusion process of compounds in microflow, making it more effective compared to two or three-phase extraction. This technique has been successfully applied in the separation of TCM [100]. This novel technology also matched the analysis of drug metabolites from biological samples. After adjusting the pH of both the donor and acceptor phases, model analytes were enriched and recovered from human urine within a 10 min extraction period [101].

Detecting medical constituents, microchip capillary electrophoresis, tandem appropriate detectors such as contactless conductive detectors, is an alternative analysis method that achieved the fast and reliable quantification of arecoline from *Semen Arecae* [102]. The designed chips also showed satisfactory pressure endurance, and researchers had constructed liquid–liquid extraction chips that matched the HPLC system and trace volume of biological or environmental samples containing the drug [56, 103]. Microfluidics with a particular channel or chamber were commonly connected to different physiochemical detectors, especially mass spectrometers, which met the analytical requirements for chemical or biological drugs. ZipChip, combined with capillary electrophoresis and native mass spectrometry, was a novel analytical platform for bio-macromolecule drugs, which achieved fast separation and detection of monoclonal antibodies (e.g., Rituximab, Trastuzumab, and Bevacizumab) and its charge variant profile without time-consumption pretreatment procedures [104]. Besides, benefiting from the utilization of capillary, paper-based micro devices achieved the objectives of separating, pre-concentrating of plasma from whole blood [105], which facilitated real-time drug monitoring in rural areas with poor medical conditions and a lack of equipment.

Microfluidic chips minimized the chemical compounds analysis system by functional micro channels and chambers, optimizing the utilization efficiency of raw materials, including crude herbs, solvent, especial biological samples [99, 100]. The diverse local structures on a chip can be designed as distinct functional regions, facilitating integration and engineering capabilities [106, 107]. Functional units on a microfluidic chip enable the real-time evaluation of endogenous and exogenous compounds during drug treatment procedure involving proteins/cells/organs [84, 108]. Multiple fragile and precious analytical protocols can be operated in this platform. These features align with the imperative to clarify TCM theories, as microfluidic chips facilitate the construction of multiple cells, tissues, and organoids co-cultures, mimicking in vivo interactions among different organs.

### Cell-on-a-chip

**Fig.4:**
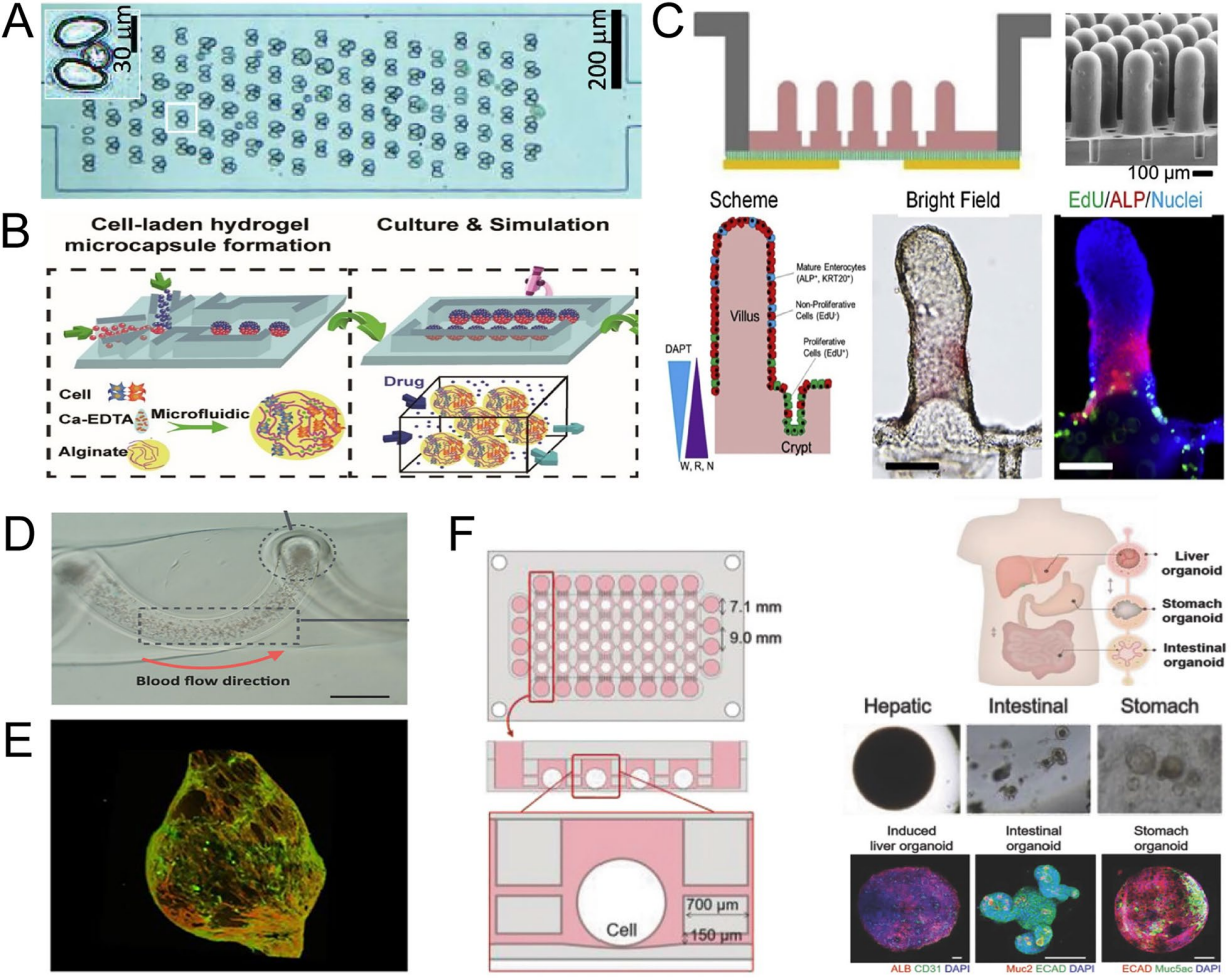
The organ/tissue mimic achieved by microfluidics. **A** Single-cell capture and analysis [110]; **B** Cells co-culture [123], Copyright, 2020, Elsevier; **C** Mimicking intestine on a chip [124], Copyright, 2017, Elsevier; **D** Biomimetic of blood vessels [80], Copyright, 2017, John Wiley and Sons; **E** Biomimetic of 3D glomerulus [125]; **F** Multi-organoids culture model [107]

The classical cell culture methods, based on microplates, have greatly advanced our understanding of biological phenomena and mechanisms. However, these conventional culture methods face challenges when investigating mechanisms at the single-cell resolution (Fig. 4A). Demonstrating the heterogeneity of cell subpopulations is difficult using average results, and the homogenization of cells or medium impedes the discovery of subtle yet significant biological information that single cells can offer. Moreover, gaining a comprehensive understanding of cell–cell interactions requires a more accessible yet robust paradigm (Fig. 4B).

The emergence of cell-on-a-chip technology has dramatically facilitated single-cell analysis through customized and flexible designs of microchannels and chambers. At the microscale, the physical properties of cells can be explored. For instance, the varying weights of different cells can be utilized to separate white blood cells from cancer cells by creating a centrifugal force gradient [109]. Cell capture is the foundation for single-cell analysis, and functional device design strategies on chips include probabilistic and deterministic designs. The latter encompasses versatile shapes and functionalized microstructures in customized chips. Single-cell trapping is often achieved by employing grooves of varying numbers and specific sizes, enabling more accessible analysis of single-cell metabolites and high-throughput measurements such as proteomics [110, 111]. Furthermore, cell-on-a-chip technology has provided a convenient means to observe and validate intercellular interactions (e.g., cellular metastasis, cascade secretion of insulin) among different cells through flexible and physiologically mimicked co-culture patterns [61, 112]. With low sample consumption and high throughput capabilities, microfluidic chips are conducive to real-time assays of cellular physiopathology, thereby accelerating the discovery of significant cell responses to microenvironmental alterations [113]. Additionally, microfluidics could enhance drug toxicity evaluations on cells [114], indicating that mimicking cellular microenvironments using microfluidics will further improve drug evaluation systems.

### Organ/organoids-on-a-chip

The development of biological affinity materials has made it possible to culture organs on chips [115]. Organ-on-a-chip, a result of integrating biology with microtechnology, has been used to simulate crucial aspects of human physiology [116]. Drawing upon our anatomical and physiological understanding of tissues and organs in animal bodies, in vitro organ culturing has been achieved by engineering different types of cells within specialized devices, including commercial instruments and laboratory-customized chips. The culture conditions are precisely controlled to maintain the physiological state of the organs. For instance, introducing a permeable endothelial barrier enables vascular flow, successfully linking multiple tissues and maintaining their structural and functional phenotypes for an extended period, up to a month [117]. The organ-on-a-chip system is capable of enabling communication between different tissues and maintaining stability over a long period of time. This provides an ideal platform for quantitative prediction of pharmacokinetics and pharmacodynamics during the pre-clinical stage [118]. Due to a shortage of monolayer cell cultures in drug screening and safety evaluation, there has been increased exploration of 3D culture techniques. Microfluidic chips simplify the multi-tissue culture process and offer more controllable properties, enabling a closer approximation of the physiological state (Fig. 4C–E). This advancement holds promise for capturing more comprehensive information regarding drug responses, including often overlooked toxicities [119].

Different from organ-on-a-chip, which performed function and application based on the mastered knowledge about organ structure and derived from the array of multiple cells, organoids-on-a-chip depended on self-organization and differentiation of homogenous pluripotent stem cells within a microfluidic chip to simulate the structural and functional characteristics of target organs [120]. Organoids generated from human pluripotent stem cells hold great promising model in the application of biomedical [121]. Blood flow is one of the critical factors of tissue development, influencing the shape and function of organs, and has the potential to be accurately simulated (Fig. 4D). Organoids-on-a-chip, which mimic blood flow through microfluidics and are generated from stem cells, provide a more comprehensive platform for manufacturing and investigating organ and tissue responses to various stimuli, including endogenous and exogenous metabolites (e.g., glucose) [122].

By utilizing microfluidic chips, it has become possible to simulate complex body structures in vitro, including airways, intestines, and other cavity-like structures [126, 127]. Building upon the successes achieved in microfluidic chip technology and the culture of cells, organs, and organoids in vitro, the concept of human-on-a-chip has emerged, gaining momentum due to its potential applications in biomedicine (Fig. 4F).

### Pharm-lab-on-a-chip

Microfluidic chips provide a versatile integration platform for the miniaturization of drug analysis and subsequent biological response assays. This is accomplished by utilizing appropriate assay technical media through macro-precise instruments or mini-sensors. The integration and automation of microfluidic chips have played a significant role in the emergence and adcancement of Pharm-on-a-chip technology [10, 128].

TCM extracts and preparations are usually taken orally, allowing them to enter the gastrointestinal tract and be absorbed by intestinal cells. Various ways have been explored to mimic the process of drug absorption, including transwell cell culture and 3D cell co-culture. However, these traditional methods using well plates have their limitations when it comes to sampling and analyzing biological samples in real-time. To overcome these limitations, developing a stomach/intestine mimic on a chip can replicate luminal flow and peristaltic-like motility in an in vitro setting [129]. In the intestine, there is drug absorption as well as efflux. Gastrointestinal cells derived from patients, incorporating personal genetic information and phenotypes, have emerged as powerful tools for drug selection using organoids-on-a-chip [130]. Intestinal cells also possess drug metabolism enzymes alongside liver cells. A novel culture pattern based on chips has showcased a more accurate representation of metabolism enzyme activity than Caco-2 monolayer cells [130], bringing it closer to real-world applications. Combining drugs is a common medical practice, but understanding its mechanism and potential harm to the body remains challenging. The flexible co-culture of gastrointestinal cells with other cell types, such as liver cells, presents an appealing approach for researchers. Using a culture solution that contains both target constituents and their metabolites simplifies the procedure, making it easier to obtain and analyze, and also improves throughput. This approach is conducive to simulating the process of drug absorption in the intestines [131].

The liver and kidney play crucial roles in drug metabolism as essential organs. It is challenging to evaluate the toxic effects of drug metabolites through conventional static well plate cultures, especially when combined with compound separation. However, using microchannels that connect different chambers, where cells/organoids/organs can coexist in vitro, has made it possible to assess potential pharmacological or toxic effects, even at trace levels of metabolites. For instance, Aflatoxin B1 exhibits no apparent toxicity on the kidney, but its metabolites harm the kidney in reverse. The hepatic-kidney chip demonstrated the indirect toxic effect of Aflatoxin B1, vividly illustrating the feasibility of a multi-organ chip in drug metabolism research [132]. By utilizing microfluidics, the transmission of trace drug metabolites becomes more efficient, eliminating time-consuming procedures such as separation and evaluation of drug metabolites. Additionally, the co-culture chip of bacteria and gastrointestinal cells, based on the intestine-chip, serves as another powerful platform for drug metabolism and screening of active metabolites.

When it comes to the distribution of medicines, it is necessary to identify the target organ of the drug. However, ethical concerns arise due to the extensive use of animal testing in drug distribution research, resulting in the sacrifice of numerous animals during pre-clinical studies. The 3D simulation of physical structures, ranging from monolayer vesicles and tissues to bio-barriers, provides a valuable approach for visualizing or predicting drug distribution in vivo. Among these structures, the blood–brain barrier (BBB) is paramount. Simulating the BBB is beneficial for understanding the effectiveness of drugs in treating brain diseases during the pre-clinical stage while saving time and resources. For instance, the BBB-glioma microfluidic chip, which consists of primary human brain microvascular endothelial cells, primary human astrocytes, primary human brain vascular pericytes, and glioma U251 cells, has been successfully employed to assess drug permeation [133].

To gain a deeper understanding of TCM combination theories about chemical compounds, multiple factors need to be taken into consideration. These factors include, but are not limited to, the following: (1) Codissolution and inhibition of compounds during the extraction process; (2) Interactions that occur during drug absorption; (3) The complex metabolic relationship of TCM constituents as substrates for drug-metabolizing enzymes; (4) Synergistic and antagonistic pharmacological effects. Among these factors, the last item is particularly important as it entails elucidating the intricate relationships between TCM and their targets (cells/tissues/organs), as well as the relationships between these targets and other entities.

**Fig.5:**
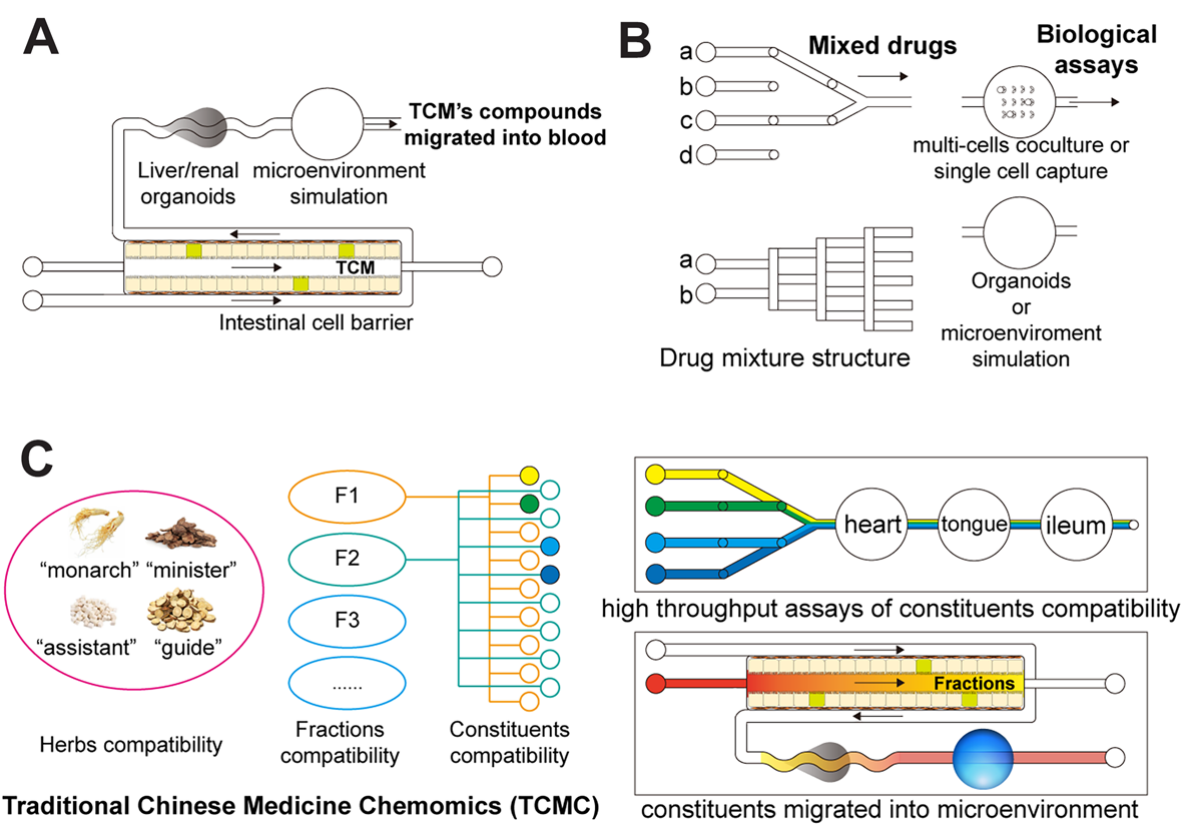
Proposed elucidation pattern of TCM compatibility theories using Pharm-on-a-chip

Microfluidic chips offer significant advantages in simulating physiological structures. By utilizing cell co-culture and external hardware-assisted molding methods, researchers have successfully constructed in vitro models of intestinal cell barrier, blood–brain barriers, and liver tissues [124, 134]. These organs/tissues play a crucial role in the absorption and metabolism of chemical components in TCM. Conventional pharmacokinetic studies typically rely on animal models to investigate the migration of TCM components into the bloodstream and the formation of metabolites. When dealing with the complex chemical composition system of TCM, studying the impact of multiple constituents on the drug process in vivo using traditional methods becomes difficult and limited in terms of feasibility. However, the integration of a biological barrier chip provides a promising solution. This technology enables high-throughput analysis of the interaction between orally administered Chinese medicines and the absorption process, without the need for live animal models (Fig. 5A).

The pharmacoefficacy evaluation of cells based on a pore plate has reached a high level of maturity. However, studying the pharmacy efficacy of multiple compounds using various combinations of TCM still poses significant challenges. A solution to this problem is offered by Pharm-on-a-chip technology, which utilizes a microtrace, high-throughput culture mode (Fig. 5B). Notably, while the scientific understanding of TCM syndrome types, such as dampness syndrome and the pattern of internal obstruction of cold-dampness, requires further exploration, the chip can simulate the microenvironment of organs and tissues. It enables monitoring of cell mechanical forces, oxygen concentration, energy metabolism, and other conditions at a microscopic level. This capability holds promise for providing insights into the tissue-level mechanisms of TCM action. Furthermore, organ interaction is a crucial aspect of TCM theory. According to classical ideas, there exist relationships between *zang-fu* organs. For example, the tongue is believed to be connected to the heart, and the heart is linked to the small intestine in terms of external and internal connections. TCM can exert regulatory effects on multiple organs. Although the general results have been recognized and theorized, there is still incomplete understanding of the specific process by which these “total effects” occur, as well as the ways in which organs cooperate. To delve deeper into this field, Pharm-on-a-chip technology can serve as a valuable platform for such research through its multi-cell/organ co-culture model (Fig. 5C).

## Utilization of microfluidic chips in traditional Chinese medicine

### Quality control

Quality control (QC) is a fundamental aspect of drugs worldwide, encompassing chemical drugs, herbs, and TCM. For drugs with known pharmacological or toxicological profiles, QC strategies focusing primarily on active or toxic ingredients have established themselves as effective and reliable methods. However, the QC system for TCM, with its complex composition and limited pharmacological understanding, exhibits unique characteristics. The evaluation items for TCM’s QC include species identification, origin locales, cultivation practices, processing methods, and manufacturing procedures. These elements are vital in guaranteeing the safety and effectiveness of TCM, microfluidics offers a novel approach for TCM’s QC (Table 1).

**Table 1:** Utilization of microfluidic chips in traditional Chinese medicine

Application	Origin	Chemical constituents	Function of microfluidics	Refs
Quality control	*Panax Ginseng*	Ginsenoside Rg_1_, Re, and Rb_1_	Successive laminar flow extraction	[100]
	*Scutellaria baicalensis* Georgi	Baicalein, wogonin, etc	Induced phase separation extraction	[136]
	*Strychnos*, *Radix Salvia Miltiorrhiza*	Strychnine, brucine, Tanshinone IIA	Three-phase extraction	[99] [98]
	*C. majus*, *M. cordata*, etc	Sanguinarine, matrine, etc	Electrochemical analysis	[160] [161] [137]
	QiShen YiQi Pills	Danshensu, salvianolic acid B, etc	Magnetic ligand fishing chip for monitoring inter-batch variation	[108]
	*Semen Platycladi*, *Pericarpium Citri Reticulataeas*, etc	Total aflatoxin	Thermal bubble pump on chip and immunoassays of toxic substances	[139]
	*Schizonepeta Tenuifolia*	Luteolin, icynaroside, rosmarinic, etc	Pharmacological evaluation of different medical parts of TCM	[135]
	*Schizonepeta Tenuifolia*	Diosmetin, luteoloside, hesperidin, etc	Cell chip for spectrum-effect relationship	[138]
Screening active compounds	*Anoectochilus Roxburghi*	Kinsenoside	3D flowing microfluidic chip	[67]
	*Macleaya cordata*	Sanguinarine, chelerythrine	Manipulating laminar flow	[143]
	*Sophora flavescens*, *Macleaya cordata*, etc	Matrine, harmide, etc	Mimicking tumor microenvironment chip	[147]
	*Tripterygium wilfordii* Hook f	Triptolide	Centrifugal microfluidic	[162]
	*Coptidis Rhizoma*	Berberine	Single-cell analytical chip	[146]
	*Mori Folium*, *Nelumbinis Folium*	Chlorogenic acid, isoquercetin, etc	Paper-based enzyme immobilized microarray	[163]
	*Oroxylum indicum* (L.) Vent	Oroxin B	Cell chip for investigating the anticancer effect	[164]
	*Scutellaria baicalensis* Georgi	Baicalein	Bacterial culture chip for drug susceptibility screening	[165]
	*Scutellaria baicalensis* Georgi, *Corydalis Rhizome*, etc	Tetrahydropalmatine, imperatorin, etc	BBB simulation chip for evaluation of the permeability of active components	[148]
Pharmacology and toxicology	*Panax Ginseng*	Ginsenosides	Cells co-culture chip for efficacy evaluation of TCM’s metabolites	[106]
	*Aconitum*	Aconitine	Chip-MS for clarifying toxicity	[159]
	*Rhodiola crenulate*	Salidroside	Mimicking and real-time monitoring of microenvironment	[157]
	*Rheum palmatum*	Emodin	Cell chip for toxicity evaluation	[166]
	*Oldenlandia Diffusa* Will	Ethanol extract	3D cells culture chip	[167]
	*Cirsium setosum* (Wild.)	Dinatin, diosmetin	Concentration gradient chip to examine medical compatibility	[144]
	Fufang Muji Granules	Multiple extract	TCM’s compatibility	[154]
	Yuxuebi capsules	Aqueous extract	TCM’s compatibility	[155]
	*Scutellaria baicalensis* Georgi, *Radix Sophorae Flavescentis*, etc	Matrine, wogonin, etc	Multiple cells co-culture chip to simulate the microenvironment of brain tumor	[133]
	*Aconitum*	Aconitine	Multiple cells co-culture particles for mimicking the microenvironment of heart	[84]

Different parts of TCM contain various chemical compositions that exhibit different efficacies. Evaluating multiple batches of herbal medicine is time-consuming and labor-intensive, and the intra-batch variation makes intra-batch comparison challenging. Employing high-throughput analysis techniques aids in identifying the efficacy of different parts of natural medicines. In this regard, microfluidic chips are instrumental in tapping into the potential of TCM resources [135]. The phase separation of complex components and selective quantification of specific chemical compositions is facilitated by laminar flow [99, 102]. For instance, an induced phase separation extraction platform was established and applied in the analysis of TCM. This platform operated within a 5 × 2 cm chip featuring microchannels measuring 100 μm in width and 40 μm in depth. According to the results, the extraction efficiency of model compounds (chlorogenic acid, epigallocatechin gallate, rutin, quercetin, santonin, and alizarin) exceeded 90%. This method were also operated in the analysis of *Scutellaria baicalensis* extract. Aglycones (baicalein, wogonin) and glycosides (baicalin, wogonoside) were separated into organic phase and aqueous phase, respectively [136]. Notably, this approach significantly reduces sample and solvent consumption to microliter quantities, with an analytical period of less than 1 min. Furthermore, the miniaturization structure on chips help simplify the analytical system and decrease data variability, making them ideal for intra-batch measurements of precious medicinal herbs (e.g., *Ginseng*) [100, 137].

The efficacy of TCM from different cultivated regions and the different medical parts of herbal plant should be evaluated objectively. Microfluidics has emerged as a valuable tool in developing rapid and simultaneous efficacy evaluation method. For example, a 4 × 4 matrix chip was designed and used to the efficacy assay of the flower, leaf, root, and stem of *Schizonepeta tenuifolia*. Human lung adenocarcinoma cell line A549 was cultured in chambers, and chambers were divided into four groups corresponding to different plant parts (flower/leaf/root/stem). Extracts obtained from these parts were pumped into cell chambers at a rate of 0.2 μL/min. After 36 h, the rates of cells apoptosis and necrosis were recorded and evaluated. The results revealed distinct effects of different plant parts, with the anti-cancer activity ranking from highest to lowest as follows: leaf, flower, root, and stem. The chip overcame the difficulty of complicated operation, poor repeatability of 96-well plate technology, and realized dynamic culture under micron scale conditions, closely mimicking real physiological conditions [135]. In addition, this platform facilitated the comparison of the pharmacological effects of *Schizonepeta tenuifolia* obtained different region such as Anhui, Henan, and Yunan province in China. Performing all procedures on a micro-scale chip also enabled convenient tracing of metabolites of natural products and monitoring of the dynamic endogenous metabolism of biological tissues, owing to the powerful integration features of microfluidics [138].

**Fig.6:**
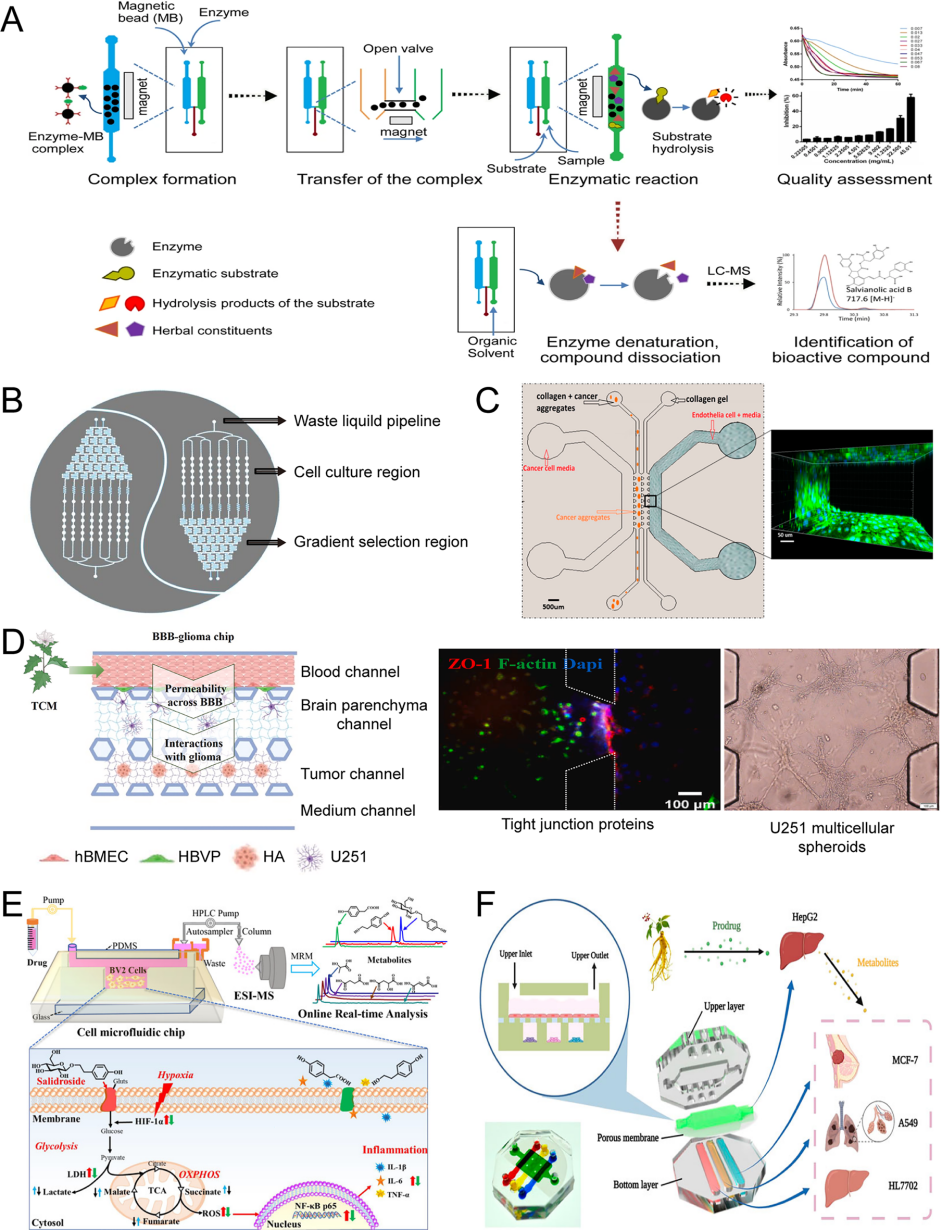
Demonstration of microfluidic chips in TCM. **A** Ligand fishing and quality control chip [108]. **B** Concentration gradient chip [144], Copyright, 2019, Elsevier; **C** Simulating of tumor microenvironment to evaluation of antimetastatic effects of TCM [147], Copyright, 2014, American Chemical Society; **D** Efficacy evaluation of TCM by biomimetic BBB chip [133], Copyright, 2023, Elsevier. **E** Endogenous and exogenous metabolites of cells were identified and monitored in real-time by the Chip-MS system [157], Copyright, 2022, American Chemical Society; **F** Cells co-culture chip for efficacy assay of TCM metabolites [106]

Facing the challenges in clarifying active compounds of TCM consisting of plant, animal, and mineral, a proposed approach for quality evaluation based on actual effects was presented. Enzyme assays are a popular method for assessing drug effects due to their ability to measure target enzyme activation or inhibition, providing insight into drug efficacy. However, enzymes are fragile, and it isn't easy to recover proteins in vitro. A minor reaction system is needed to reduce protein loss and meet usage requirements. Microfluidic chips provide an ideal solution by offering a suitable chamber for protein and small molecule incubation from TCM. In the QC of QiShen YiQi Pills (Fig. 6A), 10 μL magnetic beads and 10 μL thrombin or ACE solution were added to form enzyme-magnetic bead complexes on a chip. The micro fluid was controlled by fluidic bridge and pneumatic valve. Subsequently, 10 μL of tested sample were pump in channel and incubated with enzyme. Following this incubation period, the reaction solution was recovered, and the absorbance was measured to assess quality. Simultaneously, the enzymes were retained in the chamber and remained available for further use. The versatile chip was utilized to explore potency variations across different batches of QiShen YiQi Pills. The results showed that this approach exhibited superior discrimination ability for aberrant samples compared to chromatographic fingerprinting techniques [108].

Managing the risks associated with TCM substances is a significant field of QC. Various analytical methods, including ICP-MS, HPLC-FLD, and GC, are employed to assay heavy metals, aflatoxins, and pesticide residues, respectively. However, the determination of risk substances is typically carried out using complex instruments, which can limit the timeliness of TCM quality evaluation. Benefited from emerged surface modification, it was feasible to immobilize antibodies on a chip, leading to the development of a novel immunoassay chip that was utilized in the analysis of aflatoxins in TCMs that are rich in lipids (e.g., *Semen Platycladi*, *Pericarpium Citri Reticulataeas*, *Hellebore*, *and Semen Coicis*) [139].

### Screen of active ingredients

The effectiveness of TCM was validated by clinical application, and the predominated active constituents of TCM, including formula and single herbal medicine, needed to be clarified. Pharmacokinetics was a vital tool to describe the active compounds of TCM, and the in vivo distribution data of compounds were obtained from sacrificial animals. The biological samples were fragile, limited in volume, and non-renewable. The powerful analytical tech, especially mass spectrum, facilitated the determination and recognition of chemical constituents and their metabolites in vivo [140]. Many animals would be sacrificial under traditional sampling and determining strategies when researchers want to declare the combination mechanism of TCM by knock-in or knock-out any composition of medicinal formula.

Ligand fishing has been exploited in screening bioactive compounds, and the evaluation basis usually build on disease-associated biomacromolecules and cells [141, 142]. This promising strategy was hindered by the fragility of proteins and the high cost associated with the large volume of biological macromolecules. The consistency between in vivo and in vitro of proteins/cells has been concerned and discussed. With great biocompatibility and powerful integration potential, the micro-scale system, microfluidic chip, was found to be suitable for screening of active compounds (Table 1).

The design and application of microchannels reduced sample consumption and ensured the recovery of active macromolecule [108]. Three interconnected channels were employed in the competitive evaluation of drugs on double targets. According to the construe of chip, drug solution was directed to middle channels, while the solution containing target macromolecules was pumped to left or right channels. G-Quadruplex (HT24), dsDNA (DNA26) were used as efficacy and adverse reaction target, respectively. Based on the laminar flow, potential active components obtained from natural plant seed was screened one by one. As a result, sanguinarine and chelerythrine emerged as candidate drugs with stronger binding to HT24 than DNA26 [143]. The chip achieved the active compounds screening and toxicity evaluation simultaneously. Additionally, the Christmas tree structure integrated into the chip simplified the preparation of gradient concentration drugs. Drug candidates were mixed by laminar flow orderly and cells were treated with gradient concentration drug, thereby enhancing the throughput of active compound screening (Fig. 6B) [67, 144].

Different from homogenization evaluation, single-cell analysis contributes to precision medicine and targeted drug development [145]. In the case of micro dielectrophoresis devices, the mobility of bioparticles/particles closely correlates with the biophysical properties of the cells, facilitating the identification and trapping of special subpopulations from the whole vague population. After incubation of fluorescent zymosan bioparticles, RAW 264.7 cells were classified into different subpopulations within an EKMr range of 2.8 to 18.2 V/m^2^. Compared to the berberine treated group, RAW 264.7 cells with more engulfed fluorescent bioparticles tended to concentrate at an EKMr less than 8.2 V/m^2^. This result demonstrated that single cell phagocytic activity evaluation of TCM was accessible in microfluidic chips [146].

Compared to conventional single type cell culture or transwell cultures, multi-cell co-cultures or mimicking three-dimensional physiological structures can enhance cell-to-cell interactions, resulting in more comprehensive biological model structures and functions. Various methods have been developed to mimic the microenvironment of lesions, such as tumor spheroids, multi-cell co-culture, and organoids, to facilitate drug screening (Fig. 6B, C, F). For example, the human umbilical vein endothelial cells and cancer spheroids (40–100 μm in diameter) were placed into two adjacent channels on a chip. The collagen matrix allowed endothelial cell to form an intact endothelial monolayer and facilitated the diffusion of drug compound or cellular secretions. Twelve candidate constituents were added to the endothelial channel to resemble the drug diffusion across the capillary vessel in the circulatory system. After 36 h, the number of cell nuclei and spheroid dispersion were calculated. Nitidine and resveratrol significantly inhibited the dispersion of the spheroids, showing antimetastatic efficacy [147].

TCM formulas (e.g., kaixinsan, Yiqi Tongluo granule) have been clinically implemented as major or adjunctive compositions to treat brain disease [41]. The validation of permeability is as crucial as pharmacological assays, and microfluidics provide a conducive platform for mimicking this process. For instance, the BBB-U251 chip, a microfluidic chip that replicated the blood–brain barrier-glioma interface, was created by co-culturing human brain endothelial cells, pericytes, astrocytes, and U251 cells (Fig. 6D). This chip preserved the function of P-glycoprotein and exhibited permeability of various-sized FITC-dextran that was two to three orders of magnitude lower than that observed in Transwell systems [133]. Importantly, microfluidic chips not only simulate BBB in vitro but also provide a chamber for cell/organ culture. The co-existence of barrier tissues involved in drug metabolism and the ability to mimic lesions such as the tumor microenvironment significantly enhance the accuracy of preclinical evaluation for potential natural drug candidates [148].

### Elucidation of TCM’s compatibility

Studying the molecular-level combination mechanism is crucial for modernizing TCM, but it also poses challenges to further advancements. Many advanced theories have been proposed and applied in this field [4, 39, 44]. However, the biological evaluation and validation of complex compounds from TCM remain challenging projects. For example, the synergistic effect between different substance group of total ginsenosides and total salvianolic acid were observed a decade ago, and it is a massive consumption of labor and materials to gain an in-depth understanding of the compatibility between two groups of compounds [149–151].

Focusing on different herbal pairs was an effective way to explore the synergistic effects or potential adverse reactions in a formula, facilitating the observation of phenotype under physiological and pathological status [152]. However, the detailed molecular mechanism between chemical compounds and biological targets was urged to be elucidated. Illustrating compatibility at the herbal level is difficult. Additional dimensions, such as effective compound groups and individual molecules, are necessary [32]. Microfluidic chip has shed new light on exploring complex theory by minimizing the operation platform and providing high throughput analytical procedures, which matched the elucidation of TCM’s compatibility. Microchannels could be designed as drug mixture/treatment platforms for the laminar flow, and multiple medical compounds were utilized on a chip at different gradient concentrations or different compositions [153]. For example, twelve drug treatment groups were simultaneously designed and operated on a single chip, which optimized the concentration and ratio of the effective constituent group of TCM. The optimized compatibility ratio was validated by apoptosis test, and the efficacy of new compatibility compounds showed no significant differences between Fufang Muji Granules and its compatibility compounds [154]. This work resulted in the optimal proportions of the six groups of compounds, and it is conducive to declaring the micro mechanism of TCM’s compatibility. The high throughput platform based on microfluidic chips has shown enormous potential in the application of clarifying compatibility of TCM especially complex formulae consisting of multiple TCM [155].

### Clarification of pharmacological and toxicological efficacy

The real-time assays of the precursor/metabolites of TCM constituents and biological responses of live tissue were performed on a chip [111, 156]. Salidroside, one of the active compounds of Tibetan medicine *Rhodiola crenulate*, alleviated DFO induced hypoxia state of BV2 cells. To elucidate its efficacy mechanism, a cell microfluidic chip-mass spectrometry system was construed and utilized. Cells were cultured in chamber and treated with DFO to induce a hypoxic model. After the addition of salidroside, cell medium samples were collected every hour from outlet of microchannels, and metabolite analysis was performed using LC–MS according to the protocol. Real-time monitoring revealed changes in energy metabolic profiling. Four typical endogenous metabolites (lactate, succinate, malate, and fumarate) were confirmed by further strict quantification. Salidroside was found to reverse the dysregulation of cellular energy metabolism, thereby ameliorating DFO-induced hypoxic inflammation in BV2 cells (Fig. 6E) [157].

The in vivo metabolites of TCM are often effective in treating diseases, necessitating a detailed elucidation of their mechanisms. However, real-time trace of these bioactive compounds poses a challenge. For instance, the metabolites of some ginsenosides are bioactive constituents [158]. Recently, HepG2, A549, MCF-7, and HL7702 cells were co-cultured on a chip. HepG2 were cultured in upper chamber and other cells in separate chambers at the bottom. A porous membrane between upper and bottom chamber allowed the infusion of chemical compounds. For A549 cells, ginsenosides CK and ginsenosides Rh2 (S) exhibited antitumor effects prior to liver metabolism, with survival rates of A549 cells dropping below 40%. The effect was diminished following liver metabolism of ginsenosides. Interestingly, the growth inhibition effect of ginsenosides Rg3 (S) was observed only in the HepG2 (+) group, suggesting that hepatic metabolites of Rg3 (S) possessed anticancer activity. For MCF-7 cells, ginsenosides CK, Rh2 (S), Rg3 (S) increased early apoptosis, with Rg3 (S)’s efficacy further enhanced by liver metabolism. Through the use of LC–MS, three main metabolites (Rh2 (S), PPD, monooxygenated protopannaxadiol) of Rg3 (S) were identified and quantitative analyzed. Among them, Rh2 (S) and PPD were anticancer compounds (Fig. 6F) [106].

Toxicological efficacy could be identified and assessed using microfluidic chips. For instance, a three-phase laminar flow chip simulated the flow of micro-flow containing medical molecules and biomacromolecule. The efficacy and toxicity of four alkaloids that came from *Macleaya cordata* were assayed simultaneously by studying their binding affinity and mode [143]. Based on cell microfluidic chip-mass spectrometry system, HT22 cells were employed to investigate the neurotoxicity mechanism of aconitine. The dynamic monitoring platform demonstrated changes in metabolic profiling of amino acids and compounds related to energy metabolism. Following treatment with aconitine, HT22 cells were continuously observed for 24 h. Glutamic acid and aspartic acid were accumulated to induce neurotoxicity, and this process was followed by the energy metabolism disorder including the accumulation of lactic acid and reduction of glucose [159]. Furthermore, employing multiple channels and gases can achieved the manipulation of micro flow. This approach can generate multiple compartment cell particles and applied in the toxic evaluation of TCM. Adjacent parts allowed the interaction of different cells, offering a novel method to mimic microenvironment. For example, 3D co-culture particles (heart-on-a-particle) including HL-1 and HUVEC cells were utilized as heart models to investigate the toxic mechanism of aconitine on the heart [84].

### Application framework of microfluidic chips for TCM

**Fig.7:**
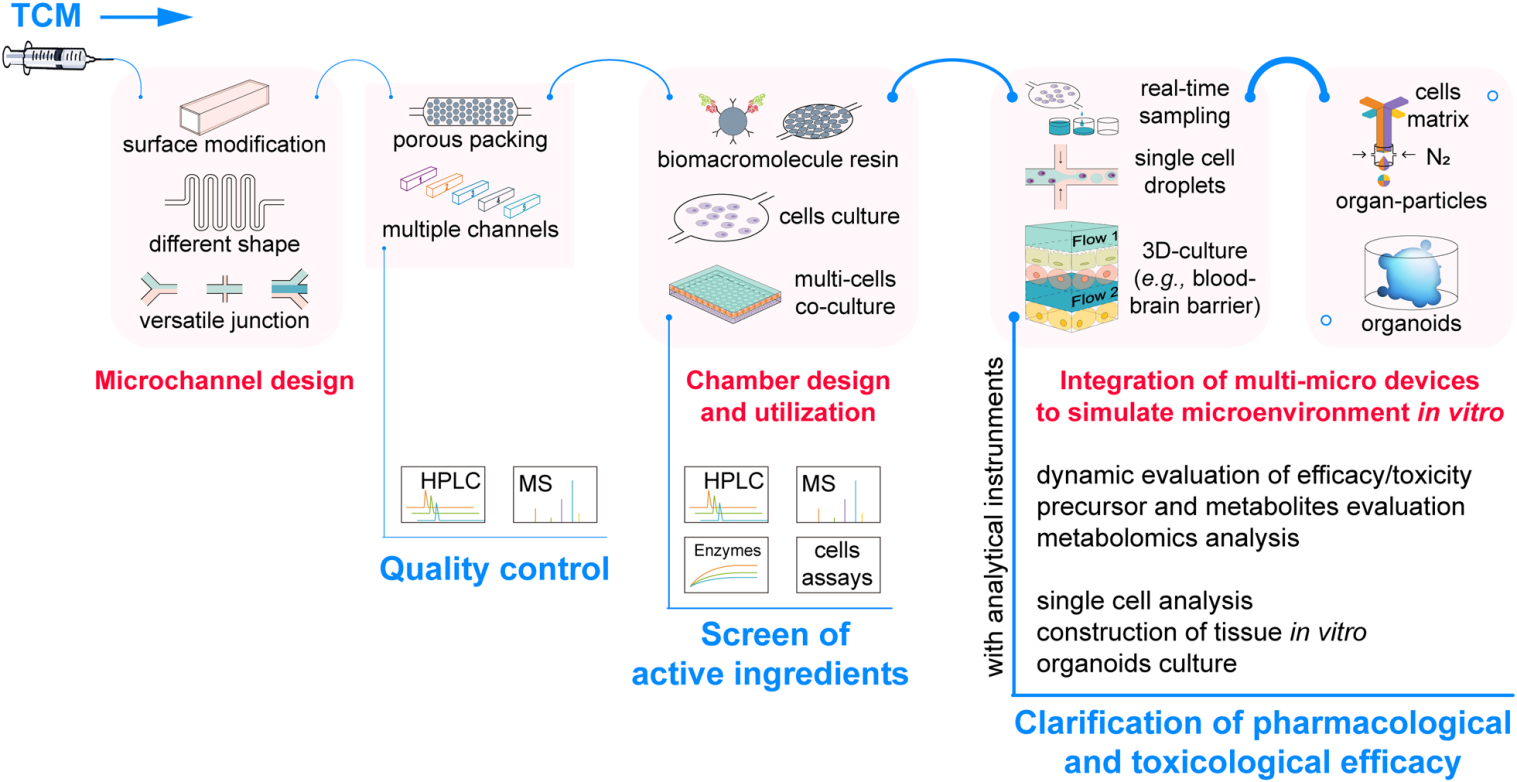
Application framework of microfluidic chips for TCM

To outline a comprehensive scheme for the utilization of microfluidic chips in TCM studies, an application framework of microfluidic chips for TCM is proposed and illustrated in Fig. 7. The design and fabrication of chips must align with the scientific objectives, and the scheme highlights key considerations during this process. In chemical compounds detection of TCM, including extraction, separation and quantitative assay, the design of microchannels is paramount. For example, the shape and number of channels on a chip influence the laminar flow dynamics, and the performance of compounds separation chips was also limited to detailed structure of junctions. The multiple channels and its downstream functional devices allows for high throughput assays on a single chip, and customized channel configurations can be implemented. Notably, the integration of HPLC–UV, HPLC–MS or other advanced detection technologies significantly enhances the sensitivity and accuracy of miniaturized total analysis systems for TCM.

In the biological evaluation of TCM, the fragility of enzymes (diseases target proteins) should be considered. The material composition of chamber influences the activity and feasibility of proteins and cultured cells. PDMS is a typical biocompatible materials used in chip manufacturing. The specific chamber design depends on the requirement of the study; for instance, circle culture chamber meet the needs of single layer cell cultures. For multi-cell co-culture chips, porous membrane or multiple layer structure are necessary in chamber construction. The flow within channels facilitates cell metabolism and material exchange between the input flow and chamber solution, thereby facilitating the screening of active ingredients in TCM.

Continuous fluid flow enables real-time sampling, and chip tandem MS system has been reported and applied in the field of drug metabolism and metabolomics. Multiple layers of channels and chambers can be form on one chip. For example, the upper channel can be used to pump flow containing TCM constituents, while intestinal epithelial cells and vascular epithelial cells can be cultured in the middle layers to partially mimic drug absorption and metabolism processes in vivo. The flow in the bottom channel can be infused with TCM metabolites, which can then be used to treat target cells.

Microfluidic chip is a powerful manufacture tool. Single cell droplets can be formed using cross junction or specific trap chambers, allowing for the acquisition of biological information distinct from homogenized analysis. Multi-stream laminar flow is beneficial for cell/organ particles and is one method for constructing organs in vitro. Microenvironment simulation is an excellent feature of microfluidic chips, and the closer the imitation, the more detailed information about efficacy or toxicity of TCM can be obtained. Hence, if necessary, complex systems containing organoids and the co-culture of different organoids are also accessible.

In summary, the manipulation of microflow is achieved through customized micro devices. This continuous fluid serves as both an input and output mechanism on the chip. Biomacromolecules, cells, or organoids can be accommodated within biocompatible chambers. The active region serves as the information exchange center on the chip. The integration of different functional regions on chips enables the execution of various analysis protocols for modernizing TCM.

## Conclusions and prospects

Inherited from microelectronics, a microfluidic chip was designed to simulate flow at the micro-scale, which was further applied in biological medicine. The microfluidic devices minimized and simplified the analytical system and showed powerful integration for its biocompatibility. Laminar flow was conducive to forming the concentration gradient of a drug, and the function of chips could be extended by surface modification or multiple-channel design. Benefiting from the permeability of gases in PDMS chips, the cell was safely cultured in a chamber. The microchannel connected the separated structures on chips, and the co-culture of multiple cells was operated easily, which breaks the limitation of static transwell mode. Culturing cells with a flowing fluid would more closely resemble the conditions in the natural body, such as shear forces and indirect communication between cells. With the discovery of cell heterogeneity, single-cell analysis has become one of the technical requirements, and microfluidic chips could capture single cells through droplet formation, functional groups on the surface, and groove structure to obtain single-cell samples. At the same time, the chip was also a suitable culture medium for organs/organoids, which provides a good research platform for microenvironment simulation.

Using research tools that closely mimic actual human conditions will expedite the process of drug development. The efficacy and theoretical system of TCM was a unique cultural heritage in China, but the explanation of the mechanism of TCM remained challenging. With the deepening of research, many theories ranging from TCMC, Chinmedomics, and Network pharmacology to TCMIP have been proposed and applied to explain efficacy mechanism of TCM. The consensus was that TCM was a multi-component, multi-target drug, and there was a complex compatibility relationship between chemical components. According to the theory of pharmacokinetics and receptor-ligand binding, the effect of TCM should be the effective constituents group entering the body, in which there were synergistic and antagonistic effects. To analyze these effects, based on studying the mechanism of action of a single compound, it is often necessary to set up different chemical composition combinations for experiments, which will be a vast workload research idea. However, the birth of microfluidic chips provided the possibility for this research. The microfluidic chip could realize multi-component, multi-gradient drug delivery and target cell and organ culture. What was particularly important is that the chip could connect a variety of cells or even multiple organs in vitro to achieve inter-organ communication. These characteristics were consistent with the combination of TCM and the theory of multiple-organ interaction.

Microfluidic chips have been applied in the separation and analysis of chemical components of TCM, which has enriched the QC system of TCM. It should be noted that TCM is a natural medicine mainly taken orally. After the administration of TCM, the internal process of constituents will be very important for pharmacological and toxicological studies, which often rely on animals, especially rodents. It has ethical implications and is limited by sample size and analytical methods. Organ/organoid chips can use human cells to construct body barriers such as intestinal cell barriers and BBB. These biological barrier chips can be used for in vitro evaluation of TCM and provide data closer to humans than animal experiments. Static cell or multicellular culture cannot monitor the migratory compounds of TCM, but a Chip-MS system can monitor the metabolites of drugs and analyze the metabolism of cells and organs at the same time, which provides a new research platform for the interpretation of the mechanism of TCM. According to TCMC, after animals, including humans, administrated TCM constituents, the effective constituents group will reach the target organs and cells along with the circulatory system and act on specific targets. Real-time sampling of target organs is conducive to explaining the mechanism of drug action. It takes work to achieve. Pharm-on-a-chip model provides multiple chambers with corresponding cells, organs, organoids, etc., that researchers can easily sample and perform analytical tests. All in all, microfluidic chips offer a powerful characterization platform for TCM multi-component, multi-target, and compatibility relations, which will further promote the modernization of TCM.

## Data Availability

Not applicable**.**

## Abbreviations

BBB: Blood–brain-barrier
GC: Gas chromatography
HPLC: High performance liquid chromatography
PDMS: Polydimethylsiloxane
QAMS: Quantitative analysis of multi-components by single marker
QC: Quality control
Re: Reynolds number
TCM: Traditional Chinese medicine
TCMC: Traditional Chinese medicine chemomics
TCMIP: Integrative pharmacology-based traditional Chinese medicine
